# Influence of DNA extraction methods on relative telomere length measurements and its impact on epidemiological studies

**DOI:** 10.1038/srep25398

**Published:** 2016-05-03

**Authors:** Julia Raschenberger, Claudia Lamina, Margot Haun, Barbara Kollerits, Stefan Coassin, Eva Boes, Ludmilla Kedenko, Anna Köttgen, Florian Kronenberg

**Affiliations:** 1 Division of Genetic Epidemiology, Department of Medical Genetics, Molecular and Clinical Pharmacology, Medical University of Innsbruck, Innsbruck, Austria; 2 Department of Internal Medicine I, Paracelsus Medical University/Salzburger Landeskliniken, Salzburg, Austria; 3 Division of Genetic Epidemiology, Institute for Medical Biometry and Statistics, Medical Center – University of Freiburg, Faculty of Medicine, University of Freiburg, Freiburg, Germany

## Abstract

Measurement of telomere length is widely used in epidemiologic studies. Insufficient standardization of the measurements processes has, however, complicated the comparison of results between studies. We aimed to investigate whether DNA extraction methods have an influence on measured values of relative telomere length (RTL) and whether this has consequences for epidemiological studies. We performed four experiments with RTL measurement in quadruplicate by qPCR using DNA extracted with different methods: 1) a standardized validation experiment including three extraction methods (magnetic-particle-method EZ1, salting-out-method INV, phenol-chloroform-isoamyl-alcohol PCI) each in the same 20 samples demonstrated pronounced differences in RTL with lowest values with EZ1 followed by INV and PCI-isolated DNA; 2) a comparison of 307 samples from an epidemiological study showing EZ1-measurements 40% lower than INV-measurements; 3) a matching-approach of two similar non-diseased control groups including 143 pairs of subjects revealed significantly shorter RTL in EZ1 than INV-extracted DNA (0.844 ± 0.157 vs. 1.357 ± 0.242); 4) an association analysis of RTL with prevalent cardiovascular disease detected a stronger association with INV than with EZ1-extracted DNA. In summary, DNA extraction methods have a pronounced influence on the measured RTL-values. This might result in spurious or lost associations in epidemiological studies under certain circumstances.

Telomeres and telomerase discovered several decades ago are considered a “protection machinery” of our genome^1^,^2^,^3^,^4^. Telomeres have been under intensive investigation due to the hypothesis that they may be responsible for aging on the cellular level and affect lifespan^5^,^6^. Many independent cross-sectional studies postulate an association of short telomere length (TL) with higher risk for various diseases such as cancer and atherosclerosis including its comorbidities. Additionally, shorter TL has been related to mortality^7^ and a variety of diseases^8^,^9^,^10^,^11^,^12^,^13^,^14^,^15^,^16^,^17^. Recently, data from prospective cohort studies including TL measurement at two different time points became available. From these studies evidence is accumulating that TL dynamics are not a one-way road with shortening over time^18^,^19^,^20^. Lengthening of telomeres can occur as well, which was observed in a large proportion (44% of 4,576 individuals) of the general population^20^. Altogether, a sinusoidal behavior of telomere length over time can be observed which decreases on average with age.

Telomere length can be measured by different methods. Widely used techniques are the absolute measurement of telomere length with restriction fragments analysis by Southern blot^21^,^22^ and the relative measurement by real-time quantitative polymerase chain reaction (qPCR)^23^. The latter is a frequently used method in epidemiological studies since much less DNA is required and it is less laborious allowing a high-throughput approach. Therefore, for all of our studies, we applied the well-established and as automated as possible high-throughput qPCR method for measurement of relative telomere length (RTL) with a high level of standardization to ensure reliable and high quality data for epidemiological studies. We generally perform RTL measurements in quadruplicate to maximize accuracy.

Comparison of TL between various studies is known to be difficult mostly due to insufficient standardization of measurements^24^. As recently reviewed^25^,^26^, inconsistencies between telomere studies might be due to different readouts such as relative values^23^, absolute values^21^,^22^, and proportion of short telomeres, but also because of differences among studied cohorts and statistical methods. The heterogeneity of results between different studies raises questions whether certain steps in the entire process of TL measurement contribute to the observed variability. We recently observed in various studies we performed that differences in the range and level of RTL measurements might be influenced by factors other than phenotypical characteristics of the investigated patients or subjects^15^. This is in line with two small studies, which both recently proposed that the results of telomere length measurement by qPCR and even Southern blotting might depend on the used DNA extraction method^27^,^28^. The central aim of the present in-depth investigation was to systematically compare the results of telomere length ascertainment by qPCR, the T/S-ratios, as a consequence of DNA extraction methods and assess its impact on epidemiological studies by four interconnected experiments.

## Materials and Methods

### Description of study samples and study designs

We performed four different experiments to clarify the influence of DNA extraction methods on the results of telomere length measurement and assess its impact on epidemiological studies.

### Experiment 1: Standardized validation experiment

EDTA blood samples were obtained from 20 volunteer blood donors from the Central Institute of Blood Transfusion and Immunology, University Hospital, Innsbruck, Austria. Written informed consent was obtained from each volunteer. Blood samples were stored at −80 °C before DNA extraction to allow for a typical laboratory process. DNA of all 20 blood samples was extracted in parallel by three different extraction methods/kits: Qiagen EZ1^®^ DNA Blood 200 μl Kit (EZ1), INVISORB^®^ Blood Universal Kit (INV) and phenol-chloroform-isoamyl-acohol extraction (PCI) (described in detail below). DNA was quantified and normalized.

### Experiment 2: Comparison of two extraction methods in the same samples of one cohort study (FHKS Study)

DNA of 307 patients of the Family Heart and Kidney Study (FHKS), an ongoing prospective multicenter cohort study of hemodialysis patients, was extracted once using the Qiagen EZ1^®^ DNA Blood 200 μl Kit and once using the INVISORB^®^ Blood Universal Kit.

### Experiment 3: Matching of two similar non-diseased control groups that applied two different DNA extraction methods

Two different random non-diseased groups recruited from the same geographical region with the same distribution of age, sex, smoking and diabetes are expected to have a similar distribution of telomere length. We therefore matched two control groups from two different studies we investigated recently. The first control group was taken from the CAVASIC (CArdioVAScular disease in patients with Intermittent Claudication) Study which is a case-control study primarily initiated to determine atherosclerotic risk factors in patients with intermittent claudication. This control group included 251 male volunteers who were recruited after an invitation in a local newspaper (Tyrol, Austria). Only individuals with presence or history of PAD were excluded^29^. The second control group consisted of subjects from the SAPHIR Study (Salzburg Atherosclerosis Prevention Program in subjects at High Individual Risk) 1:1 matched to the CAVASIC controls. Matching was performed for age (restricted to age <60 years), sex (only males), smoking and diabetes as the major determinants of telomere length. Participants from the SAPHIR Study were recruited by health-screening programs in companies in and around the city of Salzburg, Austria^30^. Since SAPHIR is recruited from a healthy working population, almost all participants were younger than 60 years. Therefore, both the CAVASIC control group and the SAPHIR Study were restricted to participants younger than 60 years, resulting in a final CAVASIC control sample of 143 individuals. For each CAVASIC control, one age-, smoking- and diabetes-matched participant was selected from the SAPHIR Study using the package “optmatch” in the statistical program R.

Different DNA extraction methods were used: the DNA of samples of the SAPHIR Study was extracted by Qiagen EZ1^®^ DNA Blood 200 μl Kit whereas the DNA of the CAVASIC Study was isolated by using the INVISORB^®^ Blood Universal Kit.

### Experiment 4: Association analysis of RTL with CVD in the FHKS Study

DNA of 307 patients as described in experiment 2 was used for this analysis. Information on prevalent CVD was available for 230 participants of FHKS of whom 119 (51.7%) had prevalent CVD.

### Ethical approval and informed consent

The examination protocols of the CAVASIC Study and the FHKS Study were approved by the Ethics Committee of the Medical University of Innsbruck. The protocol of the SAPHIR Study was approved by the Ethical Committee of Salzburg. All methods were carried out in accordance with the approved guidelines and the Declaration of Helsinki. Written informed consent was obtained from each study participant.

### DNA extraction methods

We used three different established DNA extraction methods. Whereas the first two kits and their DNA extraction principle are used in many epidemiological studies, the latter is usually no longer applied because of the toxicity of phenol. However, this method was nevertheless included because former epidemiological studies often extracted DNA using phenol. None of these methods is considered to be superior to the others and therefore, there is no natural reference or gold standard method.

### Qiagen EZ1^®^ DNA Blood 200 μl Kit (EZ1)

Genomic DNA was automatically purified by using the Qiagen EZ1 advanced Biorobot. The EZ1 instrument uses a magnetic-particle technology and performs all steps of the extraction. 200 μl whole EDTA blood is lysed and DNA is bound to magnetic beads in the presence of a chaotropic salt. Beads with the bound DNA are separated from the remaining reagents and molecules using a magnet. After a washing step DNA is eluted from the beads in 50 μl elution buffer. This method provides a pure and high-quality DNA.

### INVISORB^®^ Blood Universal Kit (INV)

Genomic DNA was isolated from 1 ml whole EDTA blood as described by the manufacturer (Stratec Molecular, Berlin, Germany). In brief, after the erythrocyte lysis the DNA was extracted and proteins were removed by digestion with Proteinase K. Precipitation of DNA by addition of a precipitation solution was followed by a washing step. Dried DNA was resuspended in elution buffer. This method provides high molecular DNA.

### Phenol-chloroform-isoamyl-alcohol extraction (PCI)

Extraction of genomic DNA was performed as with the INVISORB^®^ Blood Universal Kit with one additional step: before precipitation of DNA a phenol-chloroform-isoamyl-alcohol step was added. This extra step ensures higher purity of DNA. A disadvantage of this method is that phenol and chloroform are both toxic.

In all studies, DNA was stored at −20 °C until its usage for assessment of telomere length by qPCR.

### DNA quantification and evaluation of purity

DNA was quantified with the Tecan NanoQuant infinite M200 (Tecan Group Ltd., Männedorf, Switzerland) by measuring the absorbance at 260 and 280 nm. Purity of DNA was evaluated by detection of the absorbance 260 nm (A_260_)/absorbance 280 nm (A_280_) ratio. The OD_260/280_ ratios for the different isolation methods are shown in Table 1.

### Relative telomere length measurement by qPCR

Samples were normalized in 96-well microtiter plates and used in a singleplex, quadruplicate approach to measure the T/S-ratios (T = telomere, S = single copy gene). The T/S-ratios are proportional to individual RTL. RTL was measured with some modifications by using a quantitative real-time polymerase chain reaction (qPCR) assay, that was first described by Cawthon^23^ and that we modified as described below.

The same strategy was applied to all runs. Each qPCR was carried out in 384-well format which was horizontally segmented in two parts: telomere (T) and housekeeping gene 36B4 (S). Telomere (T) and housekeeping gene (S) PCRs were identically composed except for the primers. DNA samples were run in 15 μl reactions containing 1× Quantifast TM SYBR^®^ Green PCR master mix (Qiagen), 10 ng of DNA, 1 μM of telomere primer and 250 nm of housekeeping gene 36B4 primer, respectively. The primer sequences (5′ → 3′) were:

tel1b CGGTTTGTTTGGGTTTGGGTTTGGGTTTGGGTTTGGGTT;

tel2b GGCTTGCCTTACCCTTACCCTTACCCTTACCCTTACCCT;

36B4u CAGCAAGTGGGAAGGTGTAATCC;

36B4d CCCATTCTATCATCAACGGGTACAA^11^.

Each 384-well plate contained the standard DNA, a positive control (commercially available DNA-Human Genomic DNA, Roche) and a non-template control (NTC) in quadruplicate. The commercially available DNA was used to estimate inter-plate CV. For determination of the intra-assay CV, five different blood samples were split in 8 aliquots. Each aliquot was isolated independently by EZ1, introduced into the qPCR workflow and measured in quadruplicates. This resulted in 40 replicates (each measured in quadruplicates, equally to the study samples) in total. The resulting intra-assay CV therefore reflects the real impact on the CV of the whole workflow starting from the DNA isolation. In compliance with the MIQE (Minimum Information for publication of Quantitative real-time PCR Experiments) guidelines^31^, all intra- and interassay CV values were calculated based on the T/S ratios, not on the Ct values.

All sample transfers and dilution steps were performed with a Tecan robotic workstation with a pipetting precision for a volume of 10 μl with a CV of ~2%. Relative qPCR was carried out on an Applied Biosystems Taqman Fast Real-Time PCR 7900HT System. The thermal cycling began with the initial polymerase activation step (10 min at 95 °C) and was followed by 40 cycles of 95 °C for 15 s, 60 °C for 1 min. A melting curve analysis to verify the specificity and identity of the products was performed.

The relative quantities were determined by the efficiency correction method^32^, which does not need calibration curves and includes the individual real-time PCR efficiencies. This mathematical model calculates the ratio of a target gene (telomere) from the efficiencies and Ct-value of an experimental sample versus a standard in comparison to a reference gene (housekeeping gene). Standard DNA was the same for all experiments. To calculate PCR efficiencies of both the reference gene and the target gene PCR raw data were imported into the program LinRegPCR (version 12.5.)^33^. Based on the raw data, LinRegPCR computes efficiencies for each single replicate of each sample. The coefficients of variation of the Ct-values and the efficiency-values were assessed for each reaction to check PCR data for outliers. All single reactions showing a single CV value > 5% for either the tel PCR or the 36B4 PCR were inspected. In case that the CV deviation could be clearly attributed to one single outlying technical replicate, both Ct values of the respective replicate (tel and 36B4) were deleted. Otherwise the whole sample was excluded from analysis. For further mathematical analysis, the mean value of all efficiency-values of each gene on each plate and the mean Ct-value of the four replicates for each gene and each sample were used. Relative T/S-ratios reflect relative telomere length differences of the samples and were calculated by equation 1.


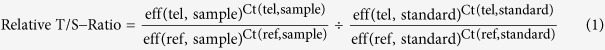


### Statistical analysis

Bland-Altman plots^34^ were used to examine the agreement between two extraction methods: the average value of both methods is plotted against the percentage difference. Proportional bias as well as 95% limits of agreement were derived using the Bland-Altman-method in both the blood-donor samples as well as in the FHKS Study. In the FHKS Study, weighted Deming regression^35^ was additionally applied to estimate both constant and/or proportional bias between methods by comparing them to the expected regression line assuming equality of both methods. In contrast to ordinary least squares linear regression, Deming regression takes errors in both variables into account. Standard errors and confidence intervals for Deming regression are derived via jackknife^36^.

To estimate the difference between the two matched groups (CAVASIC controls and SAPHIR), conditional logistic regression was applied with the matching group as stratum variable. This comparison mimics a study design, where a healthy control group is matched and compared with a clinical case group. Here, both groups are rather healthy and therefore, no difference in mean RTL would be expected especially since they are matched for variables that are known to be associated with RTL (age, sex, smoking and diabetes).

Finally, within the FHKS Study, the association of RTL with the presence of prevalent CVD is evaluated using logistic regression, adjusted for age and sex. The results of both measured RTL, isolated with INV and EZ1, were compared to each other.

For all analyses R 3.0.1 was used. The package “mcr” was applied for Deming regression analysis.

## Results

### Technical evaluation of the assay

We assessed intraplate (i.e. intra-assay) and interplate (i.e. interassay) CV of our assay using a conservative approach. In accordance with the MIQE guidelines^31^, all calculations were done based on the T/S values rater then on the Ct values, as the latter gives misleadingly low values.

To determine the intra-plate CV we did not just measure a sample multiple times, but separated the replicates already before DNA isolation. The blood of the sample was divided into in eight aliquots and each aliquot was isolated separately by EZ1. Our intra-assay CV therefore reflects the real impact of the whole workflow. This resulted in an average intra-assay CV of 7.6% (SD ± 2.8%).

**Figure 1:**
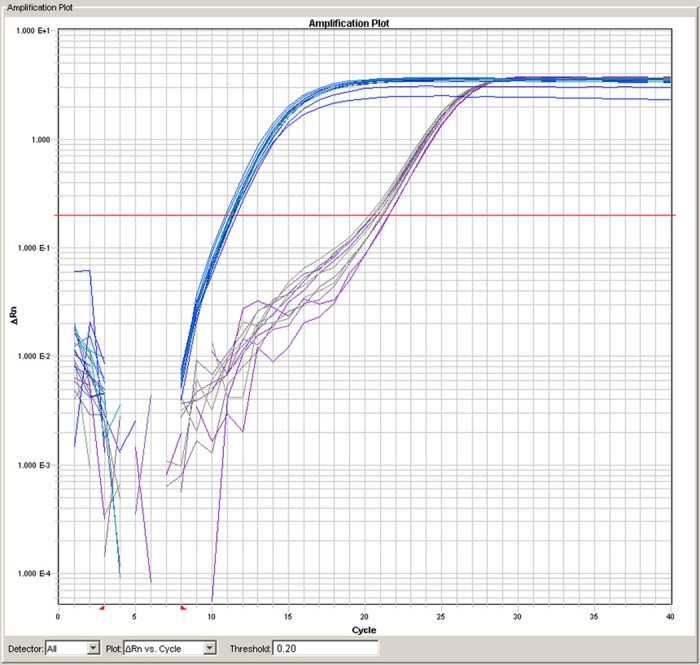
Representative amplification plot for one sample. All technical replicates of all isolation methods are superimposed. The blue curves represent the telomere product, while the purple curve represents the 36B4 product. Prior to statistical analysis the raw data was processed with LinRegPCR and inspected for outliers as described in the Materials and Methods section. The figure shows good concordance for the telomere probes over all replicates and all three extraction methods.

The inter-plate CV was determined as 10.8% using a commercially available sample present on each plate. This value is based on the presence of this DNA on each plate and does thus represent the real CV during the whole experiment, rather than the result from a dedicated preliminary evaluation experiment. The inter-assay CVs of the different studies in 64 plates were even lower and ranged from 6.08% to 7.69%. A representative amplification plot is shown in Fig. 1.

### Experiment 1: Standardized validation experiment in volunteer blood donors using 3 DNA extraction methods

**Figure 2:**
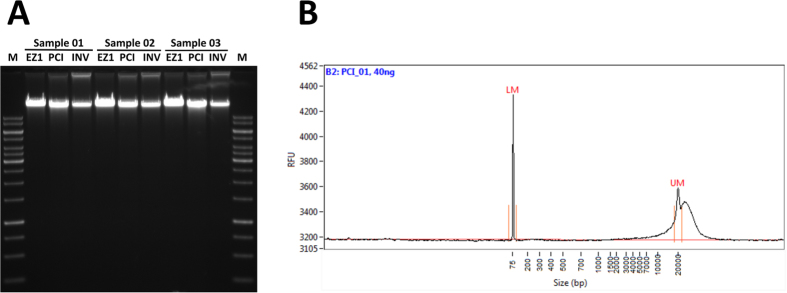
Agarose gel image and AATI Fragment analyzer trace. Panel A: Agarose gel (0.6%) image for three samples comparing the three extraction methods. The PeqGold 1 kb ladder (Peqlab Cat. No. 25–2030; 250–10,000 bp) was used as a standard. The bold fragments indicate 1 kb, 3 kb and 6 kb respectively. Panel B: A representative AATI Fragment Analyzer electropherogram. LM: lower marker at 75 bp; UM: upper marker at 20,000 bp.

All methods provided high molecular DNA of very good purity. Figure 2A shows an agarose gel for all three isolation methods for three randomly selected samples. The DNA is clearly of high molecular weight, migrating far beyond the top marker (corresponding to 10 kb). This was also confirmed by applying the same samples to an AATI Fragment Analyzer system (Advanced Analytical Technologies, Inc., Heidelberg, Germany). Figure 2B shows a representative electropherogram, showing that the DNA migrates beyond the upper 20 kb size marker. No short degradation products are visible.

The OD_260/280_ ratios and their correlation with the T/S ratio are shown in Table 1. The median OD_260/280_ ratio was >1.8 for all extraction methods. Of interest, we did not observe any correlation between DNA purity (expressed as OD_260/280_ ratio) and T/S ratio for INV and EZ1 (r^2^_EZ1_ = 0.06; r^2^_INV_ = 0.01). However, we observed a modest correlation between OD_260/280_ ratio and T/S ratio for PCI (r^2^_PCI_ = 0.45) indicating an influence of the DNA purity on the telomere measurement at least for this method. After inspection of the OD values of the PCI-extracted samples, we found three values with an OD_260/280_ ratio <1.7, which probably results from a minute carryover of organic solvent. After exclusion of these three samples, the correlation vanished (r^2^_PCI corr._ = 0.03), indicating that the DNA purity did not influence the T/S measurements. We observed essentially the same pattern also for the correlations between efficiency and T/S ratios (data not shown).

**Figure 3:**
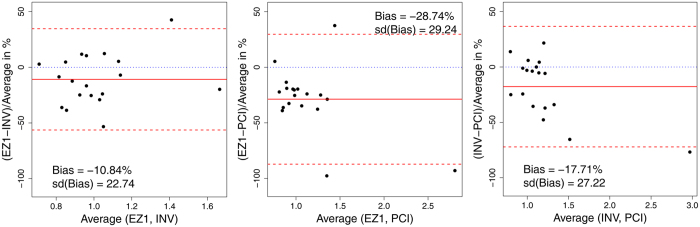
Bland-Altman-Plots comparing three DNA isolation methods in the same volunteer blood donor samples. The plots give the average of both methods on the x-axis and the percentage of difference on the y-axis. Red solid line: Mean percentage difference, red dashed lines: lower and upper 95% limits of agreement, blue dotted line: expected line of no difference; Left panel: Comparing INV with EZ1, Middle panel: Comparing PCI with EZ1, Right panel: Comparing PCI with INV.

Bland-Altman plots comparing all three isolation methods in the blood donor samples showed a considerable difference between methods with lowest RTL in DNA samples extracted with EZ1 followed by INV and PCI (Fig. 3). On average, measurements of RTL from DNA extracted by INV were ~17% lower than from DNA extracted by PCI. This difference was even more pronounced between EZ1 and PCI: RTL measured from DNA extracted by EZ1 was ~29% lower than from DNA extracted by PCI. The smallest difference was observed between INV and EZ1 with ~11% shorter RTL in EZ1-extracted samples. 95%-limits of agreement are wide and about +/−50% surrounding the average percentage difference in all three pairwise comparisons. Furthermore, the percentage difference increased with increasing average values. The three methods also differ by their variability with highest standard deviation for PCI (sd = 0.73), and considerably lower for INV (sd = 0.24) and EZ1 (sd = 0.27).

### Experiment 2: Comparison of two extraction methods in the same samples of one cohort study (FHKS Study)

**Figure 4:**
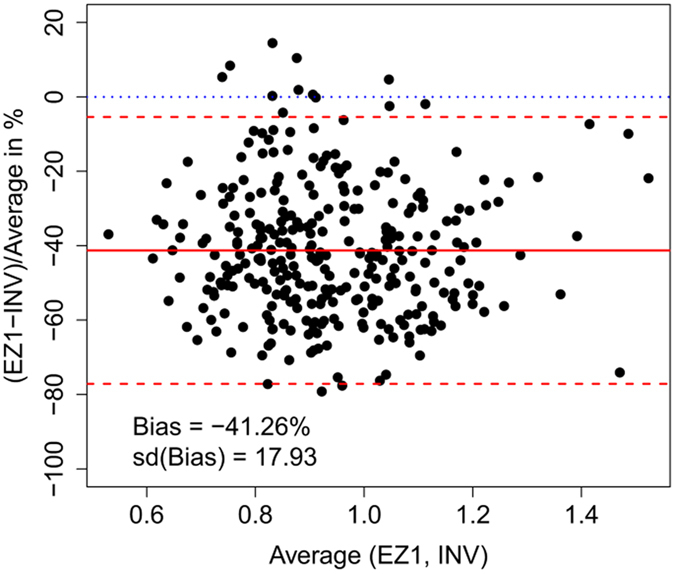
Bland-Altman-Plot comparing EZ1 and INV-isolation methods in 307 samples from the FHKS Study. The plot gives the average of both methods on the x-axis and the percentage of difference on the y-axis. Red solid line: Mean percentage difference, red dashed lines: lower and upper 95% limits of agreement, blue dotted line: expected line of no difference.

To validate findings from the standardized validation experiment in a cohort study with a larger sample size, we extracted DNA using two methods (EZ1 and INV) in DNA samples of 307 FHKS Study participants. Figure 4 shows that RTL measurements based on the EZ1-isolation method are on average 40% lower than measurements isolated by INV. Even the upper 95% limit of agreement is below 0. Therefore, it is expected that in over 95% of the samples, EZ1 measurements will be lower than INV-measurements, when both methods would be applied to the same samples.

**Figure 5:**
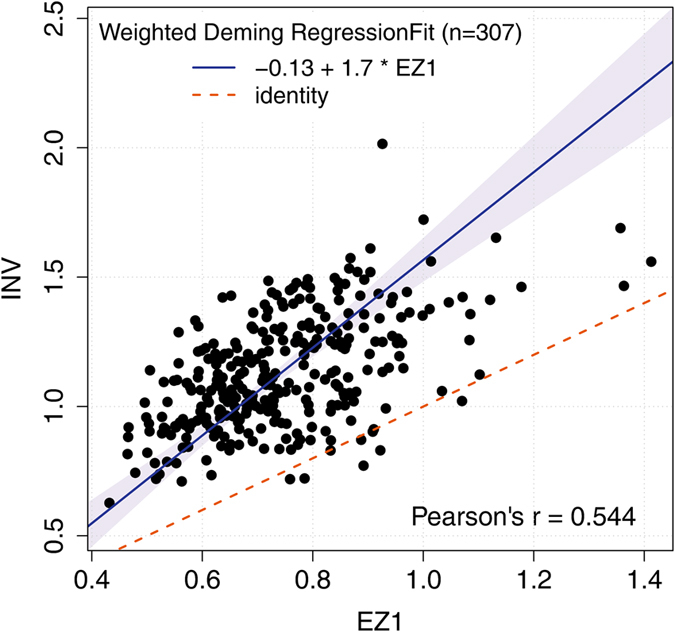
Weighted Deming regression fit of EZ1- and INV-isolation methods in 307 samples from the FHKS Study. Dashed red line: line of equality, blue solid line: Deming regression fit line with 95% confidence interval.

Since EZ1-measurements showed a lower variability than INV in the FHKS study (sd_EZ1_ = 0.16, sd_INV_ = 0.22; Ratio = 0.73), an error-ratio of 0.73 (EZ1/INV) was applied in the weighted Deming regression. It resulted in an intercept of −0.13 (95% CI −0.33–0.07) and a slope of 1.70 (95% CI 1.42–1.98), indicating a proportional bias with higher values for INV compared to EZ1 (Fig. 5). The correlation between both methods is moderate (Pearson r = 0.544).

### Experiment 3: Different DNA extraction methods in two independent matched control samples

**Figure 6:**
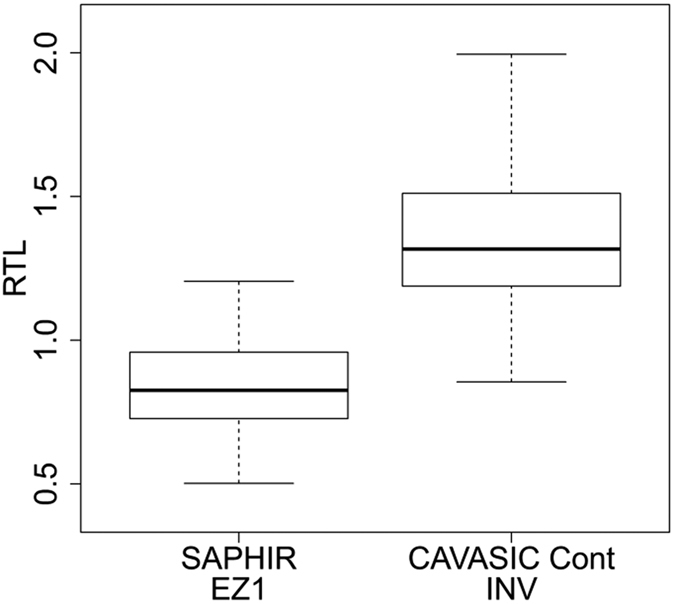
Boxplots for RTL distributions of two control groups. The two control groups are from the SAPHIR Study (EZ1-isolated) and the CAVASIC Study (INV-isolated) matched for age, sex, diabetes and smoking.

In a next step, the CAVASIC control group (n = 143) using INV isolation method was compared with the matched SAPHIR group (n = 143) using EZ1 isolation method. Both groups were 1:1 matched by age (restricted to age <60 years), sex, diabetes and smoking which are considered to be major determinants of telomere length. The distribution of age was nearly identical with median age of 48 in both groups and a range of 39–59 in the CAVASIC control group and 35–59 in the matched SAPHIR group. 8 of the 143 matching pairs were not in agreement with diabetes status and 11 not with smoking status. Although the distribution of RTL was expected to be very similar, the boxplots in Fig. 6 revealed markedly different RTL distributions between both groups with higher values for INV-isolated samples in the CAVASIC versus SAPHIR control group (mean ± sd: 1.36 ± 0.24 versus 0.84 ± 0.16).

A conditional logistic regression model of RTL on “CAVASIC-control versus SAPHIR-control-status” was expected to result in an OR of around 1 but was 5.2 for an increment of 0.1 in RTL (95% CI 2.12–12.99, p = 0.0003).

### Experiment 4: Association analysis of RTL with CVD in the FHKS Study

Finally, we performed an association analysis of RTL with prevalent CVD status within the FHKS Study. This analysis was performed twice: once using RTL measurements after DNA extraction with INV and once after extraction with EZ1. In the unadjusted model, significant associations with the presence of prevalent CVD could be detected with RTL obtained from both methods, which was more pronounced and highly significant for INV-isolated DNA: OR_EZ1_ = 1.25 (p_EZ1_ = 0.0106) and OR_INV_ = 1.32 (p_INV_ = 2.85 × 10^−5^) for each decrease of RTL by 0.1. Adjusting for sex did not change the results. Adjusting for age changed the results based on EZ1-extraction considerably and less for INV (OR_EZ1_ = 1.07, p_EZ1_ = 0.469; OR_INV_ = 1.22, p_INV_ = 0.0005).

Furthermore, Bland-Altman-analysis was performed stratified for CVD status and age: percentage bias between both methods does not depend on CVD status, (percentage bias = −44% and −41% for non-CVD and CVD, respectively), nor on age (percentage bias = −40% and −44% for participants <median and ≥median of age, respectively).

## Discussion

We systematically compared the impact of different DNA extraction methods on relative telomere length measurement by qPCR in different experimental setups. We used four different but overlapping and complementary experiments which resulted in two main findings: first, the two widely used DNA extraction methods based on either a magnetic-particle technology (EZ1) or a salting-out method (corresponds to INV) result in major differences and a rather moderate correlation of the downstream following measurement of relative telomere length. Second, ignoring these differences in the comparison of results from epidemiological studies that applied different DNA extraction methods or switching DNA extraction methods over the course of the same study might give rise to incorrect conclusions or biased associations.

The difference in RTL measured with two different DNA extraction methods in a large number of 307 samples was quite high and in line with the other experiments we conducted: RTL was roughly 40% lower when measured with EZ1 compared to INV-extracted DNA. Furthermore, the variance of RTL measured in EZ1-isolated DNA was smaller than the one from INV-isolated DNA. Usually, RTL measurement methods are standardized and quality-controlled within but not necessarily between laboratories. For example, our laboratory measures RTL in quadruplicates, other laboratories measure in duplicates or triplicates. Although RTL is possibly influenced by various factors such as inhibitors or cell population composition, diseases or life style factors (e.g. smoking), our results make it highly plausible that in the applied setup the differences observed are a result of the DNA extraction method. It is currently not clear whether it is the severity of DNA damage or other alterations during the extraction procedure that vary by extraction method. Currently, it cannot be determined which extraction method is the ‘right and appropriate’ one. We can only conclude that the basic material, namely the DNA, and thereby the method how it is extracted is one of the main contributors of differences in RTL values.

It can be excluded that the observed differences are introduced by the method of telomere length measurement itself. Recently, Cunningham *et al.* demonstrated a larger telomere length (as determined by Southern Blot) in DNA samples isolated by salting-out (corresponds to INV) compared to DNA isolated by silica columns^27^. DNA isolation by silica columns to some extent resembles the EZ1 method, as EZ1 uses silica coated magnetic beads as solid phase. This phenomenon is therefore observed for absolute and relative telomere length measurements.

An effect of DNA extraction methods on telomere range has also been recently observed by Denham *et al.* who compared two different silica column types to a salting-out approach^28^. Interestingly, they observed longer telomeres in DNA from silica columns. The results are, however, only partially comparable to both Cunningham *et al.*^27^ and to our results. While Denham *et al.*^28^ used a non-commercial salting-out protocol, both we and Cunningham *et al.*^27^ used commercial salting out kits, which provide high quality DNA. Indeed, the salted-out DNA of Denham *et al.* showed significantly lower purity than silica-isolated DNA^28^. Accordingly the authors postulate that the telomere length differences might be due to variations in qPCR kinetics caused by contaminants.

This is in line with our observations for the PCI-isolated subset, where we observed an impact of even single outlier in DNA purity. In the PCI sample, three single samples with an OD_260/280_ ratio < 1.7 already created a modest correlation (r^2^ = 0.45) between T/S ratio and OD_260/280_ ratio in the whole PCI group. The correlation vanished after exclusion of these three samples. This highlights the need of a very strict review of DNA purity and consequent exclusion of samples, which do not meet purity thresholds. This issue is very important, since the complete removal of even minute rests of organic solvents is less critical for standard SNP genotyping experiments. It might therefore not always be granted due to the mostly “SNP genotyping-oriented” design of most genetic epidemiological sample collections.

The major and burning question is whether the observed influences of the DNA extraction methods on RTL values have consequences for epidemiological studies. We conclude from our experiments that it is of utmost importance that all samples within one study are analyzed with the same DNA extraction method. This was clearly demonstrated by the first three of our four experiments, which demonstrated pronounced differences in RTL measurements when different DNA extraction methods were used on the same samples. Differences across studies that are introduced by the different extraction methods are supported by the matching experiment we carried out. This experiment mimics an often applied approach in epidemiology that matches an already existing control group to a newly recruited case group. In our experiment both matched groups were typical control groups from a similar geographical area and the same ethnical origin that were matched for major determinants of RTL (age, sex, diabetes and smoking status). One would expect to see no major differences in mean RTL values and distribution between the two control groups. However, the RTL was about 38% lower in the EZ1-isolated group compared to the INV-isolated group, which corresponds to an OR of 5.2 (p = 0.0003). This was surprising and likely introduced by the different extraction methods. Assuming the one group is a control group and the other one is a case group it would strongly depend on the method used for DNA extraction whether longer or shorter RTL would be reported for the case group. If there are indeed differences in RTL between cases and controls these could be strengthened or even vanish depending again on the DNA extraction method used in cases or controls. Therefore, the clear recommendation is to use the same DNA extraction method in the groups investigated at least as long as RTL is measured. In general, treating case and control group as similar as possible to avoid bias is one of the central propositions in epidemiology. However, no one probably would have expected that the DNA extracting method itself would have such a tremendous influence on the downstream molecular analysis. Many studies even do not mention the method of DNA extraction since it is believed to have no influences on the results for most of the molecular analysis such as genotyping and sequencing.

One might argue to be on the safe side when the same extraction method is used throughout one study and that the same results should be found when the analysis is made with either the one or the other method. However, an equally important observation was made from the association analysis of RTL with CVD within the FHKS Study: against expectations, we observed discrepancies in the associations when comparing results based on either EZ1- or INV-isolated RTL measurements. Whereas EZ1-isolated DNA results yielded no association of age-adjusted RTL with CVD, INV-isolated DNA resulted in highly significant odds ratios. From our data, however, it cannot be concluded which result reflects the truth. Usually, it is expected that a high measurement error in a reasonable-sized study does rather diminish an association than cause a false positive finding^37^, if the measurement error is non-systematic. In this case, the picture seems to be more complicated. Figure 5 (Deming regression) indicated a proportional bias with even more increasing INV-based-values for increasing EZ1-based values. Such a proportional bias alone, however, would not lead to differential association results. Such a difference can only be expected, if there is a systematic error which depends on the outcome itself or on confounding factors. More precisely, from the differences seen it could be expected that differences between INV- and EZ1-extracted RTL measurements depend on both the CVD status as well as on age. However, stratified Bland-Altman-analysis could not show differential bias depending on CVD status or age. Any dependence on other confounding factors cannot be excluded. Although no explanation can be given, the differential association results remain, which would probably lead to an enthusiastic publication in one case but would find its way into the drawer in the other case.

Our finding is in line with and extends observations by Cunningham *et al.*^27^ who systematically screened published studies on telomere length and the association with cancer risk. They showed that the majority of affinity-based studies (as EZ1) did either detect no or only a small effect whereas salting-out DNA approaches (as INV) suggested stronger association of RTL on cancer risk.

Eisenberg *et al.* very recently demonstrated a well-positioning effect in (monochrome) telomere qPCR and calculated a power reduction corresponding to −16% if well-positioning correction is not performed^38^. While these findings are clearly important, we believe that our conclusions are not significantly influenced by this observation. The DNA samples from all three extraction methods in our experiments were positioned in the same way concerning the wells on the plate. Therefore, differences between the methods have to be rather caused by issues related to the DNA extraction rather than well-positioning effects.

It should be stressed that we steer clear of a judgment which extraction method is the appropriate one and which results reflect the truth. However, we emphasize that our results by directly comparing RTL results of differently extracted DNA are important and contribute to an accurate epidemiological study setup in the highly investigated telomere biology field.

Major strengths of our study include standardized data acquisition concerning DNA extraction as well as RTL measurement. In contrast to the only former study which investigated the influence of DNA extraction on RTL values, we had four different experimental setups and a large study sample available with phenotypical data extracted by two extraction methods.

## Conclusion

Our results revealed considerable differences in RTL values and their association with study outcomes depending on the extraction method applied. Thus, the DNA extraction method can possibly influence the conclusions drawn from epidemiological studies of RTL. This indicates that maintaining the same method within one study is of high importance. Although it was not possible to elucidate the chemical and/or biological causes, the major importance of possible epidemiological consequences was illustrated. Therefore without a recommendation of the ‘appropriate’ extraction method, we emphasize the paramount significance of caution and awareness of DNA extraction and telomere length measurement in epidemiological research. This example clearly shows that the same DNA derived from the same individual by different methods might be different in some ways and this difference can have tremendous influences on the results found.

## Additional Information

**How to cite this article**: Raschenberger, J. *et al.* Influence of DNA extraction methods on relative telomere length measurements and its impact on epidemiological studies. *Sci. Rep.* **6**, 25398; doi: 10.1038/srep25398 (2016).

## Figures and Tables

**Table 1:** Descriptive data of the OD 260/280 ratio for the three extraction methods and their correlation with the T/S ratio.

	Median	Interquartile range	Min.	Max	r_Pearson_	r_Pearson_^2^
EZ1	1.85	0.04	1.80	1.89	−0.25	0.06
INV	1.89	0.08	1.82	2.07	0.11	0.01
PCI	1.84	0.03	1.44	1.89	−0.67	0.45
PCI after removal of outliers	1.84	0.02	1.70	1.89	−0.17	0.03

The PCI isolation showed three samples with an OD 260/280 ratio < 1.7.

Therefore, for the PCI isolation method, the results are shown both with and without these three samples.
